# Prevalence and Genomic Characterization of *Brucella canis* Strains Isolated from Kennels, Household, and Stray Dogs in Chile

**DOI:** 10.3390/ani10112073

**Published:** 2020-11-09

**Authors:** Nicolás Galarce, Beatriz Escobar, Eduard Martínez, Natalia Alvarado, Gabriela Peralta, Phillip Dettleff, Jessica Dorner, Víctor Martínez, Consuelo Borie

**Affiliations:** 1 Departamento de Medicina Preventiva Animal, Facultad de Ciencias Veterinarias y Pecuarias, Universidad de Chile, 8820808 Santiago, Chile; ngalarce@ug.uchile.cl (N.G.); beatrizescob@gmail.com (B.E.); martinezeduard64@gmail.com (E.M.); natalia.alvarado.i@ug.uchile.cl (N.A.); gabriela.peralta@ug.uchile.cl (G.P.); 2 Programa de Magíster en Ciencias Veterinarias, Facultad de Ciencias Veterinarias y Pecuarias, Universidad de Chile, 8820808 Santiago, Chile; 3 Laboratorio FAVET-INBIOGEN, Departamento de Fomento de la Producción Animal, Facultad de Ciencias Veterinarias y Pecuarias, Universidad de Chile, 8820808 Santiago, Chile; satryl@veterinaria.uchile.cl (P.D.); jdorner@ug.uchile.cl (J.D.); vmartine@uchile.cl (V.M.); 4 Escuela de Medicina Veterinaria, Facultad de Recursos Naturales y Medicina Veterinaria, Universidad Santo Tomás, 3473620 Santiago, Chile

**Keywords:** *Brucella canis*, canine brucellosis, canine infertility, qPCR, whole-genomic sequencing, zoonosis, stray dogs, genomic characterization

## Abstract

**Simple Summary:**

Canine brucellosis caused by *Brucella canis* is a zoonotic disease that is considered the main infectious cause of infertility and reproductive failure in dogs worldwide, particularly in Latin America. Reports of *B. canis* infection in people have increased, especially in people who work with dogs and their owners, where the adoption of stray dogs poses a public health risk. Thus, this study determined the prevalence of infection in kennels, household, and stray dogs in the Metropolitan region, Chile, recording the genomic characteristics of the isolated circulating strains. Our results demonstrate that the infection is widespread in the three canine populations analyzed, with no differences in age or sex, and most of the infected animals do not show clinical signs or bacteremia. Furthermore, the high genetic similarity of the isolated strains suggests a common transmission route throughout the country. This study corroborates the need to implement official strategies for the control and prevention of *B. canis* infection, including sanitary, diagnostic, and educational measures under the One Health vision.

**Abstract:**

Canine brucellosis caused by *Brucella canis* is a zoonotic disease that causes reproductive alterations in dogs, such as infertility, abortion, and epididymitis. This pathogen is especially prevalent in South America, and due to the lack of official control programs and the growing trend of adopting dogs it constitutes a public health risk that must be addressed. The aim of this study was to determine the prevalence of *B. canis* infection in kennel, shelter, and household dogs and to characterize the genomic properties of circulating strains, including *ure* and *vir*B operons and *omp*25/31 genes. Samples from 771 dogs were obtained, and the infection was detected by blood culture and/or serology in 7.0% of the animals. The complete *ure* and *vir*B operons and the *omp*25/31 genes were detected. Interestingly, we found different single-nucleotide polymorphisms (SNPs) in some of the analyzed genes, which could mean a change in the fitness or virulence of these strains. This study provides further evidence about dogs as a source of *B. canis* strains that can infect people. This also highlights the need to implement official control programs, including the mandatory testing of dogs, especially stray dogs, before adoption.

## 1. Introduction

Canine brucellosis is an illness caused mainly by *B. canis* and is a worldwide neglected zoonosis [1], although other species within the genus, such as *B. suis*, can also infect dogs and generate a similar disease [2]. The disease is considered to be the main cause of reproductive failures in dogs and an important cause of economic losses in breeding kennels due to abortions, stillbirths, and sperm abnormalities [1]. Non-reproductive manifestations such as discospondylitis, meningoencephalitis, and glomerulonephritis are also described, but are less common [3]. Transmission among dogs occurs mainly venereally, but also by the conjunctival and oronasal routes by contact with abortion products, vaginal secretions, milk, seminal fluids, and urine [4].

Since its discovery in 1966 in the USA [5], *B. canis* has been isolated worldwide [6]; in South America, it is considered endemic, with a seroprevalence of up to 35% [7], and specifically in Chile it ranges between 1% and 8.9% [8,9]. Even though this bacterium shows a low virulence in people, cases have been reported in HIV-infected patients [10,11], endocarditis in an adult [12], and disease in children under 4 years old [13,14,15]. Additionally, both the World Health Organization [16] and the World Organization for Animal Health (OIE) [17] point out brucellosis as meriting mandatory notification in humans and animals, respectively, although the OIE does not specify *B. canis* in its list.

*B. canis*, as well as the other species within this genus, lacks well-known bacterial virulence factors such as exotoxins, proteases, fimbriae, and capsule [18]. Thus, its virulence lies primarily in the ability to survive and multiply inside macrophages and dendritic cells [19,20], as well as modulating the host immune response. A key tool in this process is the expression of a type IV secretion system (T4SS), encoded by the *vir*B operon [18]. Other virulence factors exhibited by *B. canis* are the immunogenic outer membrane proteins (Omps), where the Omp25/31 family has been related to its invasiveness [21]. Additionally, urease activity is particularly important in human pathogenesis, allowing its survival in acidic environments such as the stomach [22].

Added to the above, all *Brucella* species are highly genetically related to each other, sharing at least a 98% genomic identity in their core genome [23]. Unfortunately, the scarce isolation of *B. canis* strains has prevented a detailed analysis of interaction with the hosts and their population dynamics in different geographical areas, where the characteristics of virulence and phylogeography can be compared. Nevertheless, in recent years some studies have addressed the genomic differences in the circulating *B. canis* strains in China [24,25,26], Brazil [27], Colombia [28], and the USA [29,30]. Thus, by using highly discriminatory techniques such as multiple locus variable-number tandem repeat analysis (MLVA), whole-genome sequencing (WGS), and whole-genome single-nucleotide polymorphisms (wgSNP), these authors have determined the existence of polymorphisms associated with adaptation to the host and the local environment [25,29]. In this context, it has been reported that these strains exhibit a high genomic homology over time, but with differences according to the geographical area of origin [25,30]. In addition, these studies have allowed to trace possible geographical routes of dissemination and sources of infection in outbreaks [29], and to associate certain genomic patterns with the fitness of the pathogen [28]. Among these genomic differences, mutations in some virulence genes, such as *omp*25, which could be related to host environmental factors, co-evolution processes, and the effectiveness of developing vaccines, have been identified. Therefore, the global comparison of genomic patterns of circulating *B. canis* strains becomes essential to improve epidemiological knowledge and knowledge of their evolutionary origin, as well as for the evaluation of public health risk.

Considering that *B. canis* strains circulating in several geographical areas exhibit some genomic differences, this study aimed to detect the infection with *B. canis* in breeding kennel, shelter, and household dogs in the metropolitan region, Chile, and also to characterize the genomic properties of the circulating strains in order to assess the potential impact of this pathogen on public health and to elucidate the genetic variation and microevolution of this pathogen.

## 2. Materials and Methods

### 2.1. Sample Collection

Dogs were sampled with prior institutional (permit code 18131-VET-UCH) and signed owner consent in the Metropolitan region in the capital region of Chile. A total of 771 samples were obtained through cephalic or jugular venipuncture, where 143 blood samples were collected from household dogs, 178 were collected from dogs from seven breeding kennels, and 450 were collected from dogs from eleven shelters during the 2018–2019 period. Sample size was calculated considering that, in the Metropolitan region, Chile, there is an estimated dog population of 1,188,468 individuals, with 394,716 registered owned dogs and a population of 274,580 of dogs that roam free [31], with a reported seroprevalence of 8.9% for household dogs [9] and 10% for stray dogs [32], with a confidence level of 95% and an α error of 5%. From animals over one year of age, without antimicrobial therapy during the previous four weeks, not pregnant or in lactation, at least 4 mL of whole blood was collected by veterinarians in Vacutainer^®^ Heparin Blood Collection Tubes (Becton, Dickinson & Co., Franklin Lakes, NJ, USA) and at least 1 mL of blood in Vacutainer^®^ Serum Blood Collection Tubes (Becton, Dickinson & Co., Franklin Lakes, NJ, USA). All the dogs were clinically examined at the time of blood collection, primarily for signs suggestive of canine brucellosis. Among these signs, we looked for lumbar pain, lameness, vaginal discharge, orchitis, epididymitis, scrotal dermatitis, testicular atrophy, uveitis, and enlargement of the lymph nodes, as well as abortion, reproductive failure, stillbirth, and infertility records. After collection, all the samples were immediately refrigerated and transported to the laboratory within 4 h.

### 2.2. Serological Samples Processing

All blood samples collected in serum tubes were centrifuged at 5000× *g* (Labofuge 200, Marshall Scientific, Hampton, NH, USA) for 10 min to separate serum from clotted blood. Next, the presence of antibodies against *B. canis* was determined by counterimmunoelectrophoresis (CIEF), with LPS-R of *B. ovis* as an antigen, and was performed at 200 V and 30 mA for 90 min [33]. Serum previously obtained from a bitch experimentally inoculated with the *B. canis* str. RM 666 was used as a positive control [34].

### 2.3. Whole Blood Samples Processing

All blood samples collected in tubes with anticoagulant were analyzed in a class 2A biosafety cabinet (Heal Force Safe 1200, Shanghai, China) with prior institutional biosecurity permission (permit code 114-VET-UCH) by means of microbiological culturing according to Alton et al. [35] and Keid et al. [36]. Briefly, 4 mL of whole blood was incubated in 40 mL of pH 7 tryptic soy broth (Becton, Dickinson & Co., Franklin Lakes, NJ, USA) supplemented with sodium citrate 2% *v/v* (Merck^®^) at 37 °C for 30 days. Every seven days, 100 µL were plated onto *Brucella* agar (Becton, Dickinson & Co., Franklin Lakes, NJ, USA) plates supplemented with cycloheximide (100 mg/L, Merck^®^, Darmstadt, Germany), bacitracin (25,000 IU, Merck^®^, Darmstadt, Germany), and polymyxin B (6000 IU, Merck^®^, Darmstadt, Germany), and each plate was incubated at 37 °C for at least 72 h. For each plate with bacterial growth, preliminary identification was performed through Gram staining and agglutination with monospecific sera anti-A (*B. abortus* str. 1119-3) and anti-R (*B. canis* str. RM 666) obtained from our collection. All suspicious colonies were cultured individually on *Brucella* agar plates (Becton, Dickinson & Co., Franklin Lakes, NJ, USA), as described above, for further identification.

### 2.4. Molecular Identification of B. canis

All presumptive *B. canis* strains were subjected to real-time PCR (qPCR) to confirm their identity. Thus, five colonies per plate per strain were suspended in 500 μL of sterile nuclease-free water and boiled for 15 min at 100 °C. A total volume of 200 µL of lysated bacteria per sample was used to purify DNA using the NucleoSpin^®^ Plant II kit (Macherey-Nagel^®^, Düren, Germany) following the manufacturer’s instructions. The concentration and quality of extracted DNA was determined by a Nanodrop spectrophotometer with EPOCH equipment (BioTek, Winooski, VT, USA) and stored at −20 °C until further use.

The purified DNA was used to perform the qPCR analysis using forward (5′-ACGAACACAAGGGCCAATAC-3′) and reverse primers (5′-GGACGGCTACAAGATCGAAG-3′), previously described for *B. canis* [37]. This qPCR was performed using a final volume of 10 µL with 5 µL of KAPA SYBR FAST qPCR Master Mix (Kapa Biosystems Inc., Wilmington, MA, USA), 0.5 µM of forward primer, 0.5 µM of reverse primer, and 4 µL of sample DNA. Cycling conditions included a 3 min denaturation (95 °C), followed by 40 amplification cycles (95 °C 5 s, 60 °C 45 s), and were performed on a Rotor-Gene Q real-time PCR cycler (Qiagen, Hilden, Germany), and the results analyzed by the Rotor-Gene Q series software (Qiagen, Hilden, Germany), determining the cycle threshold (Ct) values. The samples were considered as positive for the presence of *B. canis* if they had a Ct value lower than 35 cycles. A standard curve was generated using a 10-fold dilution series of DNA with six points, in triplicate, using nuclease-free water as a negative control, the blood DNA of a non-infected dog as an internal negative control, and DNA from *B. canis* SCL strain (access number: NZ_LGAQ00000000.1) as a positive control. All the qPCR assays were carried out complying with the Minimum Information for the Publication of Quantitative Real-Time PCR Experiments guidelines [38]. Additionally, and in order to determine the presence of the pathogen directly in blood, all the samples from seropositive or bacteriologically positive dogs were processed as described above.

### 2.5. WGS of B. canis Strains

#### 2.5.1. Library Preparation and Sequencing

A total of 1 ng of DNA was used to perform sequencing libraries using the Nextera XT DNA Library Prep kit (Illumina^®^, San Diego, CA, USA) according to the manufacturer’s protocol. The length of the libraries was determined by capillary electrophoresis (Advanced Analytical Technologies, Santa Clara, CA, USA) using the High Sensitivity NGS Fragment Analysis kit (Advanced Analytical Technologies, Santa Clara, CA, USA). Libraries were quantified using the KAPA Library Quantification kit (Kapa Biosystems Inc., Wilmington, MA, USA) using the Rotor-Gene Q series software (Qiagen, Hilden, Germany), following the manufacturer’s protocol. Libraries were sequenced on a Miseq platform (Illumina^®^, San Diego, CA, USA) using a v3 300 kit with 2 × 75 bp paired ends.

#### 2.5.2. Filtering, Assembly, Annotation, and Comparison of Bacterial Genomes

The quality of the reads was assessed using the FastQC software (Babraham Institute, Babraham, Cambridge, UK). Subsequently, raw reads were trimmed with the Fastx tool kit (Cold Spring Harbor Laboratory, Cold Spring Harbor, NY, USA) to remove low-quality reads, remaining adapters, and short-length sequences (<50 bp). *De novo* assembly was performed using the CLC Genomics Workbench v.7.0.3 software (Qiagen, Hilden, Germany). The posterior scaffolding was performed using the MeDuSa platform [39]. Each scaffold was annotated independently using the prokaryotic genome annotation service RAST [40]. The annotated genomes of all strains were simultaneously aligned and compared using the SEED Viewer module [41], with the genome of the *B. canis* SCL strain as a reference (PRJNA289097).

With the sequencing reads of the *de novo* assemblies, an analysis to identify variants between the sequenced strains against the SCL genome (PRJNA289097) was performed. The Snippy software [42] was applied, using the sequencing reads to identify variants in haploid genomes with the following parameters: minimum mapping quality = 60, minimum coverage of variant site = 15, and minimum proportion for variant evidence = 0.9. The graphical alignment was performed with the Jalview 2.11 software (The Barton Group, University of Dundee, Dundee, UK) [43]. Additionally, we looked for specific variations in the *vir*B1-12 operon, urease operon (*ure*D, *ure*E, *ure*F, *ure*G; urease subunits α, β, γ), and outer membrane proteins (*omp*25, *omp*31) encoding genes.

### 2.6. Statistical Analyses

Prevalence obtained by serology and/or blood culture was treated as a binary trait and analyzed using logistic regression in order to estimate the probabilities of risk given sex and origin (household dogs, kennel dogs, and stray dogs). The probabilities conditional on significant effects were obtained using the following model: px = 11 + e − (bo + b1x), where p(x) is the probability of success given the effect (sex and origin), bo is the intercept, b1 is the regression coefficient for each effect considered, and x is the level of the effect. All these analyses were performed using R software v3.5.0 (https://www.R-project.org) (The R Foundation, Vienna, Austria).

## 3. Results

### 3.1. Detection of Infection in Sampled Animals

From the 771 samples analyzed, the infection was detected by culture and/or serology in 7.0% (n = 54) of the animals, isolating the pathogen in 1.3% (n = 10) of them, with a seropositivity of 6.6% (n = 51). Of the 10 bacteriologically positive dogs, six corresponded to household dogs and four to stray dogs. Table 1 shows the epidemiologic characteristics of these bacteremic dogs (n = 10). On the other hand, of the seropositive dogs (n = 51) 27 corresponded to stray dogs, 16 to household dogs, and 8 to breeding kennel dogs. Among these seropositive animals, three of them had negative blood cultures.

The infected animals corresponded to 31 females and 23 males, with ages varying from 1 year to 13 years. Most animals did not show clinical signs, but in those who did the most frequently presented were discospondylitis and lumbar pain. Table S1 shows the epidemiologic characteristics of all infected dogs (n = 54). Additionally, of the seven kennels analyzed, three of them harbored at least one infected animal, while 10 of the shelters harbored at least one infected animal. Overall, the probability of infection was higher for household dogs (8%) than stray (5%) and kennel dogs (2%). No significant effects were obtained for sex and age.

### 3.2. Detection of B. canis by qPCR in Whole Blood

The results of the qPCR from blood of bacteriologically positive dogs showed amplification across all the samples (Table S2), showing consistency with the blood culture, with Ct values between 13.8 to 33.2, evidencing that the bacterial load present in blood varied individually. No amplification was detected in seropositive but bacteriologically negative dogs. Additionally, the standard curve for the set of primers was in optimal range, with an R^2^ > 0.999 and efficiency of 98% (Figure S1). Additionally, a unique amplification product was observed for the reaction, as can be observed in the melting curve analysis (Figure S2).

### 3.3. Whole-Genome Sequencing of Isolated B. canis Strains

Seven strains were subjected to WGS (9, 45, 119-1, 119-2, 124, 128, and 301), and a total of 19,627,877 reads were obtained, with an average of 2,803,982 high-quality reads per genome for the *de novo* assembly of each strain. The average coverage of sequenced strains was 100×, with an average genome size of the assembled genomes of 3,277,025 bp and an average GC content of 57.2%, presenting similar values to those of the Chilean SCL strain (Table 2). Regarding the annotation of the genomes, an average of 3395 coding sequences was annotated (Table 2).

A high similarity in the sequenced genomes compared with the SCL strain was detected. The complete *vir*B operon, urease operon, and *omp*25 and *omp*31 genes were found in all the sequenced strains. However, the variant analysis showed some differences between the sequenced strains and the SCL strain. Interestingly, we found three different SNPs (average coverage of 22×) leading to a synonymous mutation in strains 9, 45, 124, and 128. Thus, one of them was detected in the *vir*B4 gene of strain 45, another in the *ure*G gene of strains 124 and 128, and an SNP in the hypothetical protein BKD02 in strain 9. Another SNP in the methionine ABC transporter ATP-binding protein (MetN) was identified in strains 9, 45, and 124, representing a missense mutation that changed the amino acid from leucine in the SCL strain to proline (Figure S3), which could represent a neutral change in amino acid. No variants were presents in the *omp* genes. No variants were detected in strains 119-1, 119-2, and 301 with respect to SCL. Table 3 summarizes the identified SNPs, while Table 4 shows the genotypes of these SNPs.

## 4. Discussion

Several studies have addressed the seroprevalence of *B. canis* worldwide [7], but the information about the genomic characteristics of the circulating strains in Latin America is scarce [27,28,44,45]. Moreover, in Chile a limited number of studies has described the seroprevalence of *B. canis* in dogs, and none have addressed the genomic characteristics of the circulating strains [8,9,46].

Here, the low isolation rate of *B. canis* (1.3%) compared with the antibodies detection (6.6%) supports the complexity of canine brucellosis diagnosis in infected animals without bacteremia [47,48,49]. On the other hand, the presence of four bacteremic but seronegative dogs corroborated the need to use more than one diagnostic method [47,50,51]. In this context, in Hungary Gyuranecz et al. [52] observed in an infected kennel the presence of a stud dog without clinical signs but with a positive culture, and that serology turned negative one month later; in the USA, Johnson et al. [51] described a similar situation in two acute patients—a seronegative dog with discospondylitis but that was positive to intervertebral disc culture, and a seronegative bitch but with an abortion sample that was positive to culture.

It is well known that *B. canis* is detected more frequently in dogs from breeding kennels, with global seroprevalence ranging from 15% to 42.1% [50,53,54]. This could be due to the close contact of animals in these facilities and to the exchange of breeders, which facilitates the transmission and geographic spread of the pathogen [49,55]. In Chile, Pinochet et al. [56] determined a seroprevalence of 11.5% in 13 kennels in the Metropolitan region, with a prevalence of 40% in two of them. Here, we detected the infection in 4.5% of the dogs belonging to these facilities, which could indicate a higher level of care regarding reproductive management, the segregation of positive animals, and especially in performing serological tests prior to mating. On the other hand, the kennels analyzed were small, having an average of 25 animals older than one year, which makes it easier to control the associated risk factors [53].

Regarding the detection of *B. canis* infection in household dogs, the seroprevalence in some Latin American countries varies from 3.1% to 42% and is generally higher than in Europe, Asia, and North America [9,57,58,59,60,61]. In this context, although the prevalence determined here in household dogs (11.9%) is very similar to that observed in Paraguay, Colombia, and Argentina, it is lower than expected, given the total absence of specific control and prevention programs in Chile. This could be due to the greater awareness of dog owners regarding restricting their confinement and sterilizing them, thereby reducing the venereal transmission and spread of this pathogen. Here, about 50% of these of animals were gonadectomized, but this procedure only prevents sexual transmission and does not limit the transmission by other secretions such as urine [62].

Stray dogs harbored in public or private shelters currently appear as a new significant risk factor for public health due to the growing trend of adopting pets whose sanitary status prior to adoption is unknown. In these animals, the seroprevalence detected (5%) is lower than that reported in different countries such as Argentina, Colombia, Jordan, Mexico, Turkey, and the USA, with values ranging from 6.8% to 37.8% [63,64,65,66,67,68]. The registered differences are probably not only due to the sensitivity and specificity of the serological techniques used to detect anti-*B. canis* antibodies [36,69], but also to governmental, cultural, and religious differences that affect the management of these dogs [68]. In this context, the obligatory sterilization of dogs at the time of entering a shelter is a factor that may have influenced in the low seroprevalence detected in our study [70]. However, the fact that 10 of the 11 shelters presented at least one seropositive dog shows that the infection is widespread in urban areas of the Metropolitan region.

To date, few studies have been carried out on characterizing the complete genome of the circulating strains of *B. canis* in order to establish the genetic diversity and characteristics among the strains and select the best candidates for molecular epidemiology studies, especially in *B. canis* outbreaks. In this context, Di et al. [24] evaluated the genetic diversity of 29 *B. canis* isolates from China and compared them with 38 foreign isolates, detecting 57 genotypes and grouping them into five clusters, with 26 Chinese isolates presenting a mutation in the *omp*25 gene. Similarly, Vicente et al. [27] determined the existence of two genomic lineages among 53 *B. canis* isolates, grouping in lineage 1 the strains from Europe, Asia, and the USA, while lineage 2 included all South American strains. Additionally, they reported the existence of regional polymorphisms, such as the case of South America, and the circulation of different clones of *B. canis* in the same country. In the same way, Wang et al. [30] described geographic-related differences, where strains isolated from Asia and South America were grouped in the same cluster. Recently, Borie et al. [45] sequenced and characterized the complete genome of the Chilean *B. canis* SCL strain and compared its 16S gene region with that of 28 strains available at the National Center for Biotechnology Information, revealing a high similarity and suggesting a single spread route of this pathogen in South America, possibly from North America. These genomic differences could be related to their different geographical origin and also as a response to adapting to the conditions of different hosts and maintaining their fitness [44]. In the present study, of the seven sequenced *B. canis* strains isolated, three of them did not present genetic variants with respect to the *B. canis* SCL strain, while the remaining harbored some SNPs. Interestingly, three strains presented a missense mutation in the methionine ABC transporter ATP-binding protein (MetN) gene, changing the amino acid from leucine to proline. These transporters are responsible for importing and exporting relevant molecules in the cell and are present in bacterial genomes in contiguous open reading frames that share the substrate and the protein family [71]. In other *Brucella* species, ABC transporters have been directly associated with their virulence [72,73,74] or even used as a vaccine target [75]. However, in *B. canis* further studies are needed to determine the relevance of ABC systems in virulence and the possible effect of this mutation in these strains.

Regarding other virulence factors of *B. canis*, studies of their variations have become more relevant, especially characterizing those that are considered targets for vaccine development such as the *omp* and *vir*B genes [76,77,78]. In this context, de la Cuesta-Zuluaga et al. [79] analyzed the sequences of some *vir*B genes from seven strains of *B. canis* isolated from dogs in Colombia. These strains showed a high genetic similarity to each other, although SNPs and mutations were detected in the whole *vir*B operon. Additionally, these authors registered polymorphisms both between and within the breeding facilities analyzed, suggesting the circulation of more than one clone even within the same kennel. On the other hand, our genomic analysis revealed that the seven strains analyzed presented the complete *vir*B operon (1–12), demonstrating a high similarity between them and also with the *B. canis* SCL strain, although a SNP was detected in the *vir*B4 gene in one strain (strain # 45) that produced a synonymous mutation. The VirB4 plays a crucial role in the virulence of *Brucella* spp., constituting together with VirB11 an ATPases/energy center [80], so that a mutation in this gene could affect the functionality of the whole T4SS. Nevertheless, further studies are necessary to determine the functionality and expression of this secretion system in *B. canis*. The *omp*25 and *omp*31 genes sequenced are similar to the previously described SCL strain, presenting a non-synonymous mutation in the *omp*25 gene and in the *omp*31 gene, resulting in a change in protein length due to the presence of a stop codon [45]. These mutations could mean not only a change in the functionality of these proteins and therefore a change in the virulence [18,81], but also in antigenic properties, which should be considered in the development of vaccines. On the other hand, all seven strains presented the complete *ure* operon (*ure*1 and *ure*2), with strains 124 and 128 presenting an SNP in the *ure*G gene, unlike the SCL strain that presents a SNP in the α subunit [45]. Nevertheless, further studies are needed in order to elucidate the biological effect of this change.

## 5. Conclusions

This is the first study that characterized *B. canis* strains circulating in Chile at the genomic level in a region where a high number of the human population lives in close contact with dogs, both owned and unowned. Our results show that *B. canis* infection remains endemic in the Metropolitan region of Chile, being detected in household, stray, and breeding dogs, with a prevalence similar to that of other Latin American countries, which certainly constitutes a risk for animal and public health. Additionally, the circulating strains show a low genomic diversity, which could suggest a possible single spreading route in Chile. These findings are essential in order to improve the epidemiological analysis between isolated strains of dogs and people; to trace their geographical origin, especially in outbreaks; and also to determine transmission routes, altogether in the One Health concept. Moreover, the genomic characterization of certain virulence factors is a tool that can be used to improve the diagnosis of this neglected pathogen and the development of effective vaccines. Finally, although we detected a low prevalence of *B. canis* infection in only one region of Chile, our results highlight the need to implement official control programs, including the mandatory testing of dogs, especially in stray dogs prior to their adoption.

## Figures and Tables

**Table 1:** Epidemiologic characteristics of the bacteriologically and qPCR-confirmed infected dogs.

Dog Number	Origin	Age (Years)	Sex	Reproductive Status	Clinical Signs	Serology
45	Household	5	Female	Gonadectomized	None	+
119-1	Household	1	Female	Entire	Abortion	+
119-2	Household	1	Female	Entire	None	+
124	Household	7	Female	Entire	Abortion	−
128	Household	2	Male	Entire	Discospondylitis	+
301	Household	4	Female	Entire	Discospondylitis	+
6	Stray	2	Female	Gonadectomized	None	+
9	Stray	7	Male	Gonadectomized	None	−
18	Stray	1	Male	Gonadectomized	None	−
29	Stray	3	Male	Gonadectomized	None	+

**Table 2:** Genome *de novo* assembly and annotation statistics of sequenced strains of *B. canis* using *B. canis* SCL strain as reference.

Statistics	9	45	119-1	119-2	124	128	301	SCL
Genome size (bp)	3,254,281	3,250,735	3,307,435	3,253,352	3,296,208	3,284,800	3,292,366	3,284,845
GC Content (%)	57.3	57.3	56.8	57.3	57.2	57.3	57.2	57.3
N50	124,708	104,649	8754	145,362	196,611	87,181	180,729	134,581
# of Contigs	2	2	144	3	14	4	12	53
# of Coding Sequences	3281	3282	3879	3297	3356	3320	3347	3313
# of RNAs	52	50	62	49	52	51	50	49

**Table 3:** Comparative analysis of variants in the genomes of the Chilean *B. canis* isolates with the SCL strain.

	Strain Number						
	9	45	119-1	119-2	124	128	301
# of SNPs with SCL strain	2	2	0	0	2	1	0

**Table 4:** Genotypes of the identified SNPs among Chilean *B. canis* isolates with the SCL strain.

	*met*N		*ure*G		*vir*B4		Hypothetical Protein BKD02	
Strain	Genotype	Position	Genotype	Position	Genotype	Position	Genotype	Position
SCL	A	18861	G	6120	G	9679	C	117032
9	G	18861	G	6120	G	9679	T	117032
45	G	18861	G	6120	T	9679	C	117032
119-1	A	18861	G	6120	G	9679	C	117032
119-2	A	18861	G	6120	G	9679	C	117032
124	G	18861	A	6120	G	9679	C	117032
128	A	18861	A	6120	G	9679	C	117032
301	A	18861	G	6120	G	9679	C	117032

## Supplementary Materials

The following are available online at https://www.mdpi.com/2076-2615/10/11/2073/s1: Figure S1: Standard curve of qPCR amplification of *B. canis* isolated strain. Figure S2: Melt curve of qPCR amplification of *B. canis* isolated strains. Figure S3: Alignment of MetN amino acid sequences of Chilean *B. canis* strains and SCL strain. Different colors indicate different amino acid and clearer column. Table S1: Epidemiologic characteristics of infected dogs. Table S2: CT values, serology and blood culture results of dog samples. All positive samples to qPCR have Ct values under 35 cycles. All negative samples by qPCR presented in the table have no presented amplification (no Ct values).
