# Supplementary material for: Prevalence and Genomic Characterization of Brucella canis Strains Isolated from Kennels, Household, and Stray Dogs in Chile
Source: Animals (Basel). 2020 Nov 9;10(11):2073. doi: 10.3390/ani10112073 (PMC7695308; doi:10.3390/ani10112073)
Supplement: Supplementary file 1 [file animals-10-02073-s001.zip › (NG) Supplementary Materials_NG.docx]

**Supplementary Materials**

**Table S1.** Epidemiologic characteristics of infected dogs

| **Dog number** | **Origin** | **Age (years)** | **Sex** | **Serology** | **Culture** | **Reproductive status** | **Clinical signs** |
| --- | --- | --- | --- | --- | --- | --- | --- |
| 5 | Household | 1 | Female | Positive | Negative | Gonadectomized | Low back pain |
| 128 | Household | 2 | Male | Positive | Positive | Entire | Discospondylitis |
| 205 | Household | 4 | Female | Positive | Negative | Gonadectomized | Arthritis and low back pain |
| 251 | Household | 6 | Male | Positive | Negative | Gonadectomized | Discopondylitis (acute tetraparesis) |
| 265 | Household | 1 | Female | Positive | Negative | Gonadectomized | None |
| 266 | Household | 2 | Female | Positive | Negative | Gonadectomized | None |
| 284 | Household | 5 | Male | Positive | Negative | Entire | Orchitis and low back pain |
| 285 | Household | 5 | Female | Positive | Negative | Entire | Low back pain |
| 286 | Household | 1 | Female | Positive | Negative | Entire | None |
| 299 | Household | 3 | Male | Positive | Negative | Gonadectomized | Discospondylitis |
| 301 | Household | 5 | Female | Positive | Positive | Entire | Discospondylitis |
| 320 | Household | 2 | Male | Positive | Negative | Gonadectomized | None |
| 45 | Household | 5 | Female | Positive | Positive | Gonadectomized | None |
| 119-1 | Household | 1 | Female | Positive | Positive | Entire | Abortion |
| 124 | Household | 7 | Female | Negative | Positive | Entire | Abortion |
| 119-2 | Household | 1 | Female | Positive | Positive | Entire | None |
| 260 | Household | 10 | Female | Positive | Negative | Gonadectomized | None |
| 152 | Kennel | 2 | Female | Positive | Negative | Entire | None |
| 274 | Kennel | 5 | Male | Positive | Negative | Entire | None |
| 275 | Kennel | 4 | Female | Positive | Negative | Entire | None |
| 277 | Kennel | 1 | Male | Positive | Negative | Entire | None |
| 282 | Kennel | 8 | Male | Positive | Negative | Entire | None |
| 252 | Kennel | 2 | Male | Positive | Negative | Entire | Lower back pain and lameness of right forelimb |
| 255 | Kennel | 4 | Male | Positive | Negative | Entire | None |
| 256 | Kennel | 4 | Female | Positive | Negative | Gonadectomized | None |
| 6SD | Shelter | 2 | Female | Positive | Positive | Gonadectomized | None |
| 9SD | Shelter | 7 | Male | Negative | Positive | Gonadectomized | None |
| 14SD | Shelter | ND | Male | Positive | Negative | Gonadectomized | None |
| 87SD | Shelter | ND | Female | Positive | Negative | Gonadectomized | None |
| 221SD | Shelter | ND | Female | Positive | Negative | Gonadectomized | None |
| 227SD | Shelter | ND | Female | Positive | Negative | Gonadectomized | None |
| 229SD | Shelter | ND | Female | Positive | Negative | Gonadectomized | None |
| U3 | Shelter | 8 | Male | Positive | Negative | Gonadectomized | None |
| U13 | Shelter | 5 | Female | Positive | Negative | Gonadectomized | None |
| U23 | Shelter | 3 | Male | Positive | Negative | Gonadectomized | None |
| U24 | Shelter | 1 | Male | Positive | Negative | Gonadectomized | None |
| U25 | Shelter | 2 | Female | Positive | Negative | Entire | None |
| R4 | Shelter | 3 | Female | Positive | Negative | Gonadectomized | None |
| C22 | Shelter | 3 | Male | Positive | Negative | Gonadectomized | None |
| C35 | Shelter | 10 | Male | Positive | Negative | Gonadectomized | None |
| C36 | Shelter | 9 | Male | Positive | Negative | Entire | None |
| C49 | Shelter | 7 | Female | Positive | Negative | Gonadectomized | None |
| C54 | Shelter | 10 | Female | Positive | Negative | Gonadectomized | None |
| C62 | Shelter | 13 | Female | Positive | Negative | Gonadectomized | None |
| C68 | Shelter | 10 | Female | Positive | Negative | Gonadectomized | None |
| L4 | Shelter | 2 | Male | Positive | Negative | Gonadectomized | None |
| L6 | Shelter | 7 | Male | Positive | Negative | Gonadectomized | None |
| V6 | Shelter | 6 | Male | Positive | Negative | Gonadectomized | None |
| V16 | Shelter | 4 | Female | Positive | Negative | Gonadectomized | None |
| M17 | Shelter | 3 | Female | Positive | Negative | Gonadectomized | None |
| M18 | Shelter | 1 | Male | Negative | Positive | Gonadectomized | None |
| M29 | Shelter | 3 | Male | Positive | Positive | Gonadectomized | None |
| A6 | Shelter | 3 | Female | Positive | Negative | Gonadectomized | None |
| A8 | Shelter | 4 | Female | Positive | Negative | Gonadectomized | None |

**Table S2.** CT values, serology and blood culture results of dog samples. All positive samples to qPCR have Ct values under 35 cycles. All negative samples by qPCR presented in the table have no presented amplification (no Ct values)

| **Sample** | **CT (qPCR)** | **Serology** | **Blood culture** |
| --- | --- | --- | --- |
| 6 | + | + | + |
| 9 | + | - | + |
| 45 | + | + | + |
| 119-1 | + | + | + |
| 119-2 | + | + | + |
| 124 | + | - | + |
| 128 | + | + | + |
| 301 | + | + | + |
| 18 | + | + | + |
| 29 | + | + | + |
| 5 | - | + | - |
| 14 | - | + | - |
| 87 | - | + | - |
| 125 | - | + | - |
| 152 | - | + | - |
| 205 | - | + | - |
| 251 | - | + | - |
| 252 | - | + | - |
| 255 | - | + | - |
| 256 | - | + | - |
| 260 | - | + | - |
| 265 | - | + | - |
| 266 | - | + | - |
| 277 | - | + | - |
| 284 | - | + | - |
| 285 | - | + | - |
| 286 | - | + | - |


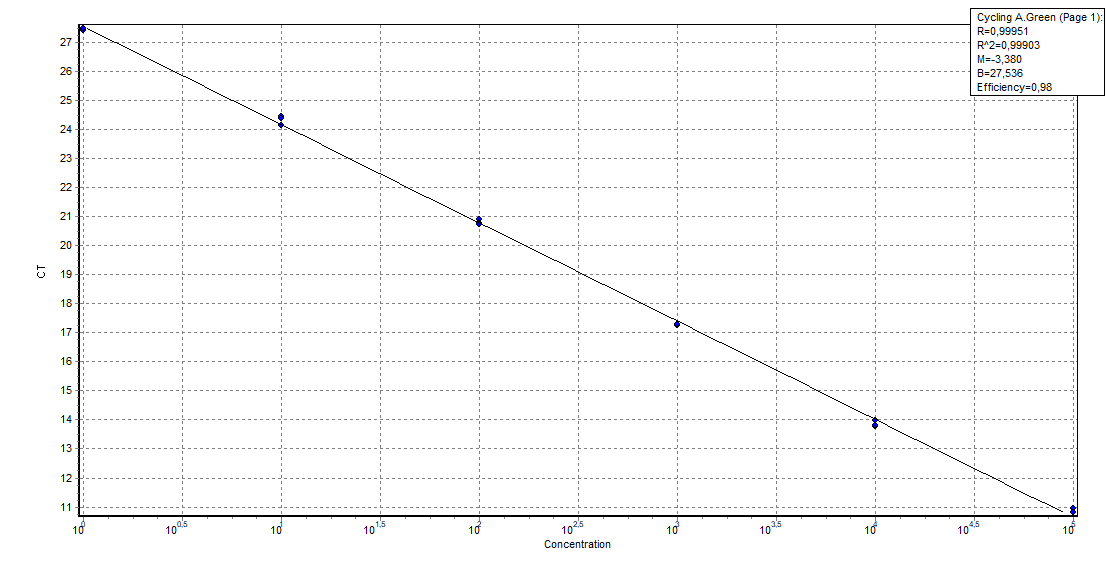


**Figure S1.** Standard curve of qPCR amplification of a *B. canis* isolated strain.

**
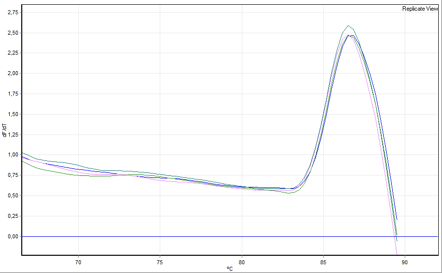
**

**Figure S2.** Melt curve of qPCR amplification of *B. canis* isolated strains.


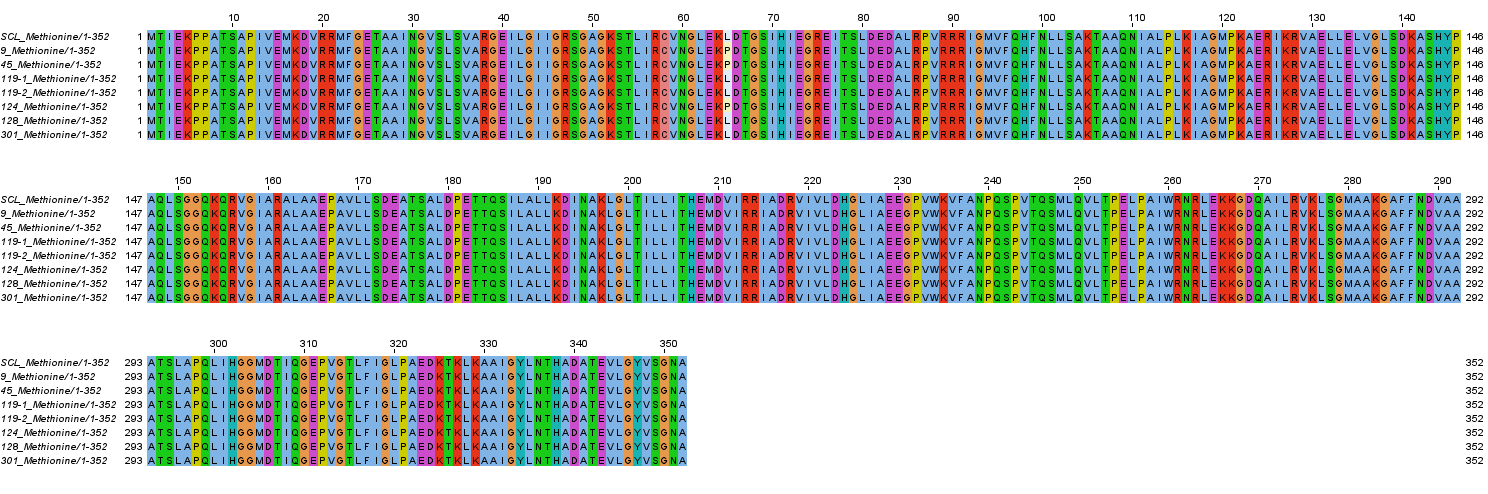


**Figure S3.** Alignment of MetN amino acid sequences of Chilean *B. canis* strains and SCL strain*.* Different colors indicate different amino acid and clearer column.
