# Supplementary figures and images for: Prevalence and Genomic Characterization of Brucella canis Strains Isolated from Kennels, Household, and Stray Dogs in Chile
Source: Animals (Basel). 2020 Nov 9;10(11):2073. doi: 10.3390/ani10112073 (PMC7695308; doi:10.3390/ani10112073)

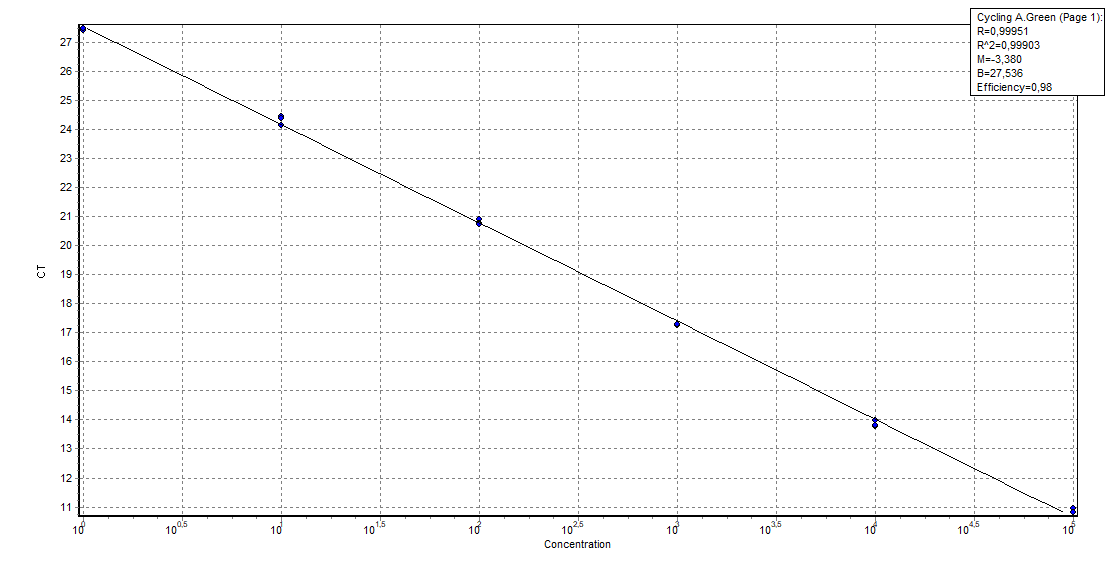

Supplement: Supplementary file 1 [file animals-10-02073-s001.zip › (NG) Fig. S1.tif]

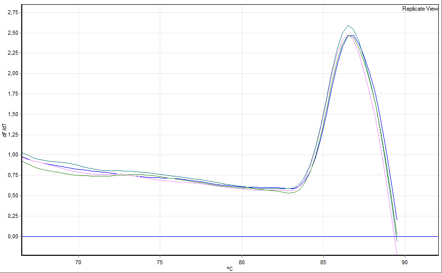

Supplement: Supplementary file 1 [file animals-10-02073-s001.zip › (NG) Fig. S2.tif]

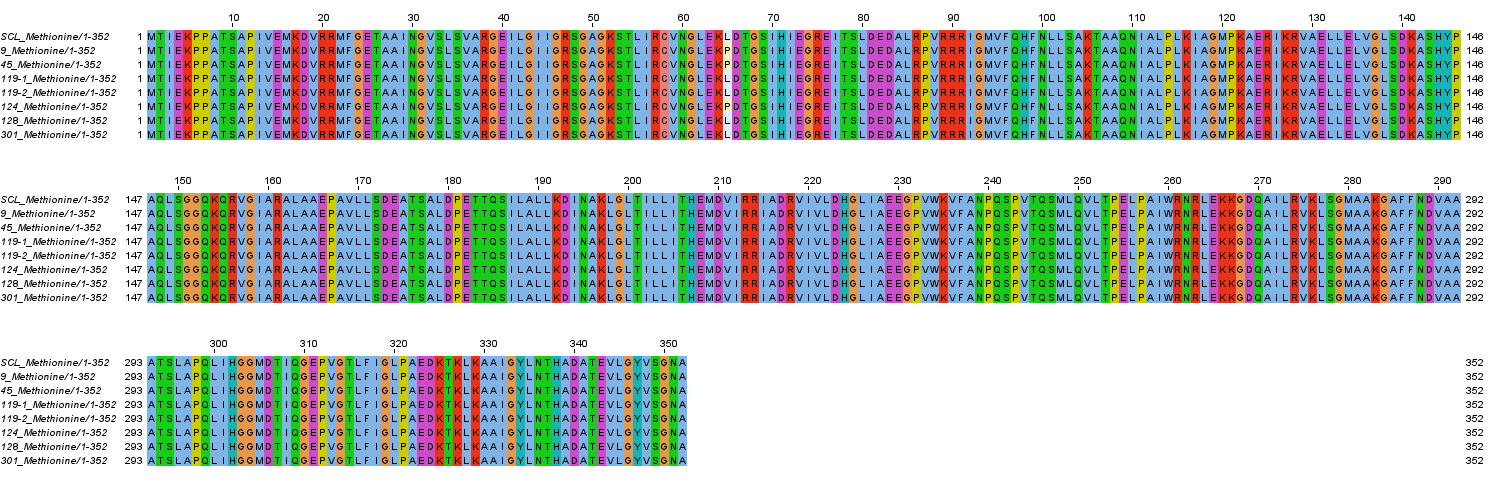

Supplement: Supplementary file 1 [file animals-10-02073-s001.zip › (NG) Fig. S3.tif]
